# Effectiveness of Arm Swing Exercise on Comprehensive Health Outcomes: A Systematic Review and Meta-Analysis

**DOI:** 10.3390/healthcare13182357

**Published:** 2025-09-19

**Authors:** Phatcharaphon Whaikid, Noppawan Piaseu

**Affiliations:** Ramathibodi School of Nursing, Faculty of Medicine Ramathibodi Hospital, Mahidol University, Bangkok 10400, Thailand; phatcharaporn.wha@hcu.ac.th

**Keywords:** arm swing exercise, systematic review, meta-analysis

## Abstract

**Background:** Arm swing exercise (ASE) is a simple and accessible form of physical activity that has been reported to reduce disease risk and enhance overall health across various populations. In alignment with the World Health Organization’s recommendations for promoting physical activity, ASE requires no specialized equipment or professional supervision. However, systematic evidence on its health benefits remains limited. **Objective:** This systematic review and meta-analysis aimed to assess the effects of ASE on body composition, glycemic control, cardiovascular health, and musculoskeletal outcomes. **Methods:** A comprehensive systematic search was conducted in four major databases, including PubMed, Embase, Scopus, and the Thai-Journal Citation Index Center, covering studies published from inception to October 2024. Articles published in both English and Thai were included. Two independent reviewers screened and selected eligible randomized controlled trials and quasi-experimental studies. Methodological quality was assessed using the JBI critical appraisal tool, and meta-analyses were performed using Stata software (version 18), presenting mean differences (MD) with 95% confidence intervals (CI). **Results:** Thirteen studies were included, comprising eight randomized controlled trials and five quasi-experimental studies, with intervention durations ranging from 5 to 24 weeks. The ASE significantly reduced waist circumference (MD = −4.76; 95% CI: −8.36 to −1.17; and *p* < 0.05), hemoglobin A1C (MD= −0.80%; 95% CI: −1.19 to −0.40; and *p* < 0.001), fasting blood glucose (MD = −17.62 mg/dL; 95% CI: −25.93 to −9.32; and *p* < 0.05), and diastolic blood pressure (MD = −9.74 mmHg; 95% CI: −17.84 to −1.65; and *p* < 0.05). The ASE showed a non-significant reduction in systolic blood pressure (MD = −3.65 mmHg; 95% CI: −10.37 to 3.08; and *p* = 0.29). Additionally, the ASE significantly increased high-density lipoprotein cholesterol (HDL-C) levels (MD = 6.96 mg/dL; 95% CI: 2.20 to 11.71; and *p* < 0.05). **Conclusions:** This study, representing the first systematic review and meta-analysis focusing on ASE, demonstrates that ASE is an effective intervention for improving body composition, glycemic control, and cardiovascular health. Given its simplicity, low cost, and broad applicability, ASE could serve as a practical public health strategy to promote health and prevent chronic diseases across diverse populations.

## 1. Introduction

Physical activity is fundamental to maintaining overall health [1], especially in older populations or people with limited mobility [2]. Regular exercise has been proven to improve various health outcomes, such as type 2 diabetes mellitus (T2DM) [3,4], cardiovascular function [5], blood pressure in people with hypertension [6], muscle strength, and mental well-being [7,8].

Arm swing exercise (ASE) is a simple, rhythmic, low-impact movement involving standing upright and swinging both arms backward and forward in a controlled manner. It is highly accessible, requires no equipment, and can be performed independently. ASE is officially endorsed in Thailand’s public health policy as a low-barrier physical activity intervention and is widely implemented through community health promotion programs due to its simplicity and adaptability across age groups [9]. ASE can also be conceptually integrated into broader physical activity frameworks such as yoga, tai chi, and analytical locomotor approaches. These modalities emphasize controlled, mindful movement, postural alignment, and neuromuscular coordination—principles central to ASE. By engaging the upper body in rhythmic motion, ASE promotes cardiovascular stimulation, core stability, and functional mobility. It is especially beneficial for individuals with limited lower limb mobility [9,10].

ASE offers a unique and often underappreciated approach to physical fitness, providing benefits that are equal to or even superior to lower extremity exercises in certain contexts. Research shows that arm swing is not merely a passive movement but actively contributes to lower limb muscle activation and coordination during walking [10]. This neuromuscular engagement supports improved gait efficiency and balance, which is especially beneficial in rehabilitation settings such as stroke recovery. In dynamic movements like vertical jumping, arm swinging has been shown to significantly increase jump height—by up to 33%—due to enhanced ground reaction forces and extended impulse duration, even though EMG activity in lower limb muscles may not always show a direct increase [11]. Additionally, during running, active arm swinging improves the rotational stability of the torso and reduces overall metabolic energy consumption, making it an energy-efficient mechanism for locomotion [12]. These findings highlight the functional importance of arm swing in promoting cardiovascular health, core stability, and movement efficiency, while also offering a low-impact, inclusive alternative for individuals with limited lower limb mobility.

ASE contributes to multifaceted health benefits, including improved balance [13], which is a crucial factor in preventing falls, especially in older adults. Fall-related injuries are a significant cause of morbidity and mortality in older populations [14,15]. Additionally, exercises enhance circulation and stimulate the cardiovascular system [16], with ASE potentially improving cardiovascular health and reducing the risk of hypertension and other cardiovascular conditions. Previous studies have also demonstrated their effectiveness in lowering hypertension [13,17]. Metabolically, physical activity helps regulate blood sugar [18] and cholesterol levels [19]; ASE is potentially beneficial for people with diabetes or those at risk of metabolic disorders.

ASE typically begins with a gentle warm-up (e.g., stretching or slow walking), followed by 30 min of rhythmic arm swinging with feet shoulder-width apart. Arms are swung backward (~60°) and forward (~30°) in a smooth, continuous motion. A cool-down phase with slow arm movements and stretching concludes the session safely.

Despite these promising indications, the evidence supporting the broad range of health benefits associated with ASE remains sparse and fragmented. Previous studies have often focused on specific populations or single outcomes, leaving gaps in understanding its overall impact. This lack of comprehensive evidence highlights the need for a systematic review and meta-analysis to evaluate the potential benefits of ASE more thoroughly. Physical inactivity remains a leading global health issue, contributing to the burden of non-communicable diseases worldwide. Accessible and low-cost exercises such as ASE may serve as practical strategies to address physical inactivity, especially in low- and middle-income countries where access to formal exercise facilities is limited. Therefore, this systematic review and meta-analysis seek to provide a clearer understanding of the efficacy of ASE in improving health and preventing disease, with the goal of promoting their broader application in health interventions.

## 2. Methods and Analysis

### 2.1. Study Design

A systematic review and meta-analysis were conducted in accordance with the Preferred Reporting Items for Systematic Reviews and Meta-Analyses (PRISMA) 2020 guidelines. The review protocol was registered with the International Prospective Register of Systematic Reviews (PROSPERO; registration number CRD42024589443).

### 2.2. Search Strategy

A systematic search was conducted in four major databases, including PubMed, Embase, Scopus, and Thai-Journal Citation Index Center (TCI), to identify studies published from inception to October 2024 by two independent reviewers. To ensure comprehensive coverage, both English and Thai-language articles were included. The search strategy focused on key terms aligned with the PICO framework: P (Population): age > 18; I (Intervention): arm swing exercise; C (Comparison): daily activities or standard care, O (Outcome): comprehensive health outcomes and specific conditions (e.g., hypertension, systolic and diastolic blood pressure, glycemic control, muscle strength, and balance).

The search strategy was developed based on the PICO framework: (“Adults” OR “older adults” OR “aged 18 years and over”) AND (“Arm swing exercise” OR “upper limb movement”) AND (“Health outcomes” OR “hypertension” OR “muscle strength”). 

### 2.3. Inclusion Criteria

This systematic review and meta-analysis specifically focus on ASE; therefore, only studies that explicitly evaluated ASE as the primary type of physical activity intervention were involved. The inclusion criteria for this review are as follows: (1) Randomized controlled trials (RCTs) or quasi-experimental studies with a control group are included, (2) participants must be adults aged 18 years or older, (3) the study must investigate ASE as the primary intervention, (4) the control group must consist of individuals receiving standard care or those maintaining their usual daily activities without performing ASE, (5) studies must report at least one relevant health outcome (blood pressure (systolic and diastolic), glycemic control (e.g., HbA1c, fasting blood glucose (FBG)), muscle strength, balance, posture, and other related health indicators), and (6) only full-text articles published in English or Thai were eligible. To maintain the quality and integrity of this review, studies are excluded if they: Are published solely as pilot studies, conference abstracts, or review articles, or lack complete and reliable data necessary for analysis.

### 2.4. Outcome Measures

The outcome measures for this review encompassed a broad range of health indicators to assess the impact of ASE on overall well-being. Glycemic control was evaluated using key markers, including HbA1c and FBG levels, which reflect both long-term and short-term blood sugar management. In terms of cardiovascular health, the analysis focused on blood pressure readings—both systolic and diastolic—as well as lipid profile components when available. Anthropometric measurements, such as waist circumference (WC), were also considered to gauge central obesity and its associated risks. Additionally, physical performance outcomes were assessed through evaluations of muscle strength and balance, serving as indicators of functional fitness. Where reported, other relevant health markers, including those related to cardiovascular function and inflammatory status, were integrated into the analysis. Together, these outcome measures provided a comprehensive framework for determining the effectiveness of ASE on diverse aspects of health.

### 2.5. Articles Selection and Data Extraction

#### 2.5.1. Study Selection Process

All retrieved publications were imported into EndNote (Version 21 for Windows, Clarivate Analytics) for deduplication and organization. After duplicate records were removed, the remaining studies underwent a structured screening process based on their titles and abstracts, ensuring alignment with the inclusion and exclusion criteria. Following this initial screening, potentially relevant studies were retrieved in full text for a more detailed assessment. Any discrepancies in study selection were discussed and resolved between the reviewers. The study selection process was thoroughly documented to ensure transparency and reproducibility. This systematic approach helped eliminate bias and ensured that only well-designed and methodologically sound studies were included in the final synthesis.

#### 2.5.2. Data Extraction

The data extraction process was conducted systematically to ensure accuracy and consistency. Initially, two independent reviewers screened the titles and abstracts of all identified studies to assess their relevance based on predefined inclusion criteria. Full-text articles of potentially eligible studies were retrieved for further evaluation. In cases where discrepancies arose, discussions were held to resolve any differences and reach a consensus.

A carefully designed and standardized data extraction framework was followed to maintain uniformity. Key information was collected, including (1) author’s name and year of publication, (2) study design, (3) country, (4) participant details such as age and sample size, (5) intervention type, (6) duration of intervention, and (7) reported outcomes. Any inconsistencies were resolved through discussion between the two authors, ensuring the reliability of the extracted data.

### 2.6. Quality and Risk of Bias Assessment

To ensure the reliability and validity of this systematic review and meta-analysis, the quality of the included studies was assessed using the Joanna Briggs Institute (JBI) critical appraisal checklists. These checklists evaluated key methodological aspects of RCTs [20] and quasi-experimental designs [21], identifying potential biases and ensuring the robustness of findings.

For RCTs, the evaluation expanded to thirteen domains to ensure the highest level of methodological rigor. This included assessing the randomization process, allocation concealment, and baseline comparability of groups. It also examined whether blinding was applied to participants, personnel, and outcome assessors to minimize bias. Furthermore, it considered whether the intervention was delivered consistently, follow-up was adequate, and any loss to follow-up was addressed. The use of intention-to-treat analysis, reliability of outcome measures, appropriateness of statistical methods, and completeness of reported results were also key factors in determining study quality.

For quasi-experimental designs, the assessment focused on nine key areas: the clarity of the cause-and-effect relationship, the comparability of intervention and control groups, and the method of group allocation. It also examined how well the study controlled confounding factors, whether outcomes were measured before or after the intervention, and the reliability of these measurements. Additionally, it considered participant follow-up, consistency in intervention delivery, and the appropriateness of statistical analysis.

### 2.7. Statistical Analysis

All statistical analyses were conducted using Stata version 18. A meta-analysis was performed to estimate effect sizes for continuous outcomes, with results reported as mean differences (MD) and 95% confidence intervals (CIs) to reflect the precision and reliability of the findings. To evaluate variability among studies, heterogeneity was assessed using the I^2^ statistic, which quantifies the proportion of variation due to actual differences rather than random chance.

Heterogeneity levels were categorized as follows: 0.0–24.9% indicated minimal heterogeneity, 25.0–49.9% suggested moderate heterogeneity, 50.0–74.9% represented substantial heterogeneity, and 75.0–100% signified considerable heterogeneity. Additionally, the χ^2^ test was applied to assess the statistical significance of heterogeneity, with a *p*-value < 0.1 considered indicative of meaningful variation across studies. We conducted a sensitivity analysis to evaluate the stability of the findings

## 3. Results

### 3.1. Articles Selection and Characteristics

**Figure 1:**
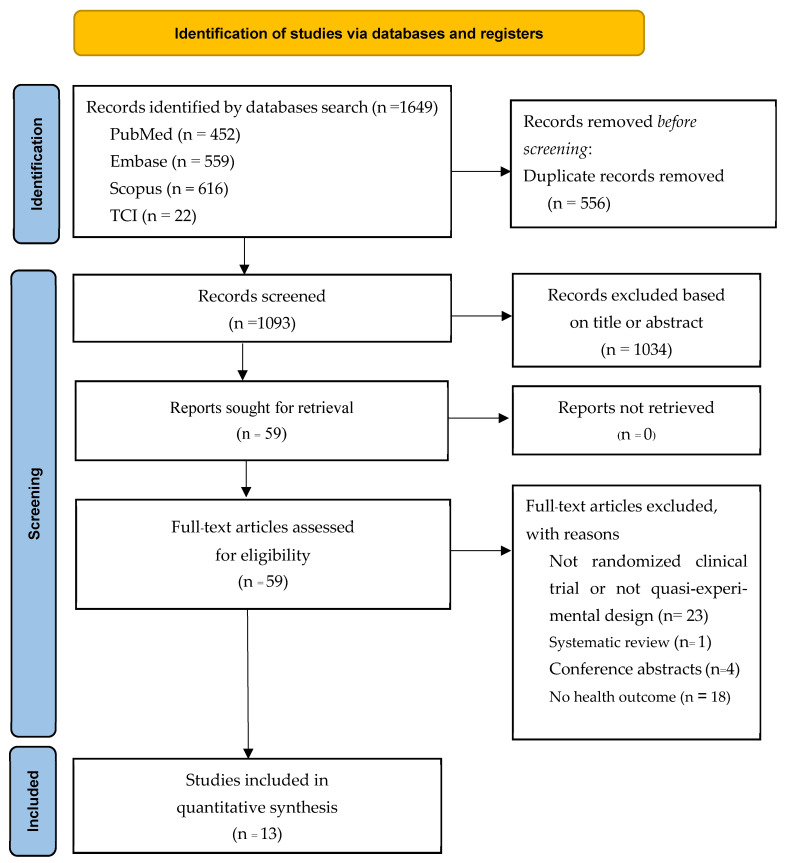
Flow diagram for a systematic review and meta-analysis.

The systematic review and meta-analysis process is illustrated in Figure 1. The study selection was conducted systematically to ensure transparency and the inclusion of only the most relevant studies. A total of 1649 records were retrieved from four major databases: PubMed (n = 452), Embase (n = 559), Scopus (n = 616), and TCI (n = 22). After duplicate records were identified and removed (n = 556), 1093 records remained for screening. Two independent reviewers screened the titles and abstracts based on predefined inclusion and exclusion criteria. At this stage, 1034 records were excluded due to irrelevance. Subsequently, 59 studies were sought for retrieval, and all were assessed for eligibility through a full-text review. During the full-text assessment, 23 studies were excluded for not being RCTs or quasi-experimental designs, 1 study was excluded for being a systematic review, 4 studies were excluded for being conference abstracts, and 18 studies were excluded due to the absence of relevant health outcomes. Ultimately, 13 studies were included in the quantitative synthesis. The entire selection process was documented using a PRISMA flow diagram, ensuring clarity and reproducibility.

### 3.2. Quality and Risk of Bias Assessment

We use the JBI checklist for quasi-experimental designs and RCTs. A critical appraisal was conducted to assess the quality of evidence from the 13 included studies, consisting of 8 RCTs and 5 quasi-experimental studies.

The assessment indicated that the quasi-experimental studies predominantly received a “yes” rating, with four of them achieving a full 9/9 score. One study had at least two “unclear” (U) ratings, while no studies received “no” (N) ratings. The key methodological aspects—clarity in cause-and-effect relationships, comparability of groups, outcome measurement consistency, and statistical analysis appropriateness—were generally rated as “low risk” of bias, ensuring strong methodological quality among these studies.

For the RCTs, the evaluation yielded a mix of “yes,” “no,” and “unclear” responses. Among them, one study achieved 11 “yes” ratings, and one study achieved 10 “yes” ratings, reflecting strong methodological rigor. The remaining six studies received between 8 and 9 “yes” ratings, with at least 2 “no” ratings each. Additionally, seven RCTs had at least two “unclear” ratings, primarily concerning allocation concealment (Q2) and blinding procedures (Q4–Q6). Across all studies, key methodological aspects—including randomization, baseline comparability, analysis consistency, outcome measurement uniformity, and statistical analysis appropriateness—were predominantly rated as “low risk” of bias, ensuring a robust study quality (Supplementary File S1).

### 3.3. Data Synthesis of Outcome Measures

The effectiveness of ASE has been demonstrated through multiple studies conducted in Thailand, employing RCTs and quasi-experimental designs. Participants ranged from 18 to 87 years old, and intervention durations varied from 8 to 24 weeks. The ASE was performed 3 to 5 days per week, with session durations ranging from 15 to 130 min. These studies collectively highlight ASE as an effective intervention for improving various aspects of health, as follows:

ASE has shown significant benefits for cardiovascular health, primarily by reducing blood pressure and improving circulatory function. Studies examining ASE interventions lasting between 12 and 24 weeks have reported notable reductions in systolic blood pressure (SBP) and diastolic blood pressure (DBP), making it a valuable non-pharmacological approach for managing hypertension. Notably, the included studies demonstrated significant improvements in blood pressure regulation [13,17,22,23,24]. Additionally, ASE has been found to enhance cardiorespiratory fitness, as evidenced by increases in the VO_2_ peak and exercise capacity following interventions lasting 5 to 8 weeks [25,26].

ASE contributes to metabolic health improvements by promoting better blood glucose regulation. Several studies have reported reductions in FBG and HbA1c, both of which are critical markers for diabetes management. Specifically, ASE interventions of 12 weeks led to significant reductions in HbA1c [27,28], while reductions in FBG were observed in studies lasting 12 weeks [23,25].

ASE has also demonstrated the potential to reduce inflammation and cardiovascular risk, with reductions observed in high-sensitivity C-reactive protein (hsCRP), as well as in pulse pressure (PP), and low-frequency (LF) power, after 12-week interventions [17,22]. Additionally, ASE has been linked to improvements in lipid metabolism and electrolyte balance, particularly through increased levels of high-density lipoprotein (HDL) and the better regulation of potassium (K^+^) and magnesium (Mg^2+^) [22]. Similarly, ASE enhanced HDL levels in participants after just 5 weeks of training [25].

In addition to metabolic and cardiovascular benefits, ASE plays a crucial role in musculoskeletal and functional enhancements. Studies have reported significant improvements in muscle strength, endurance, flexibility, and balance, which are essential for maintaining mobility and reducing fall risks. For instance, one study reported significant improvements in muscle strength and endurance after 8 weeks of ASE training [29]. Furthermore, ASE demonstrated improvements in posture, flexibility, gait, and cognitive function, in an 8-week trial [8].

Lastly, ASE has been shown to positively impact body composition by reducing key indicators of obesity and metabolic syndrome risk, including WC, waist-to-hip ratio (W/H), and body mass index (BMI). Several studies have reported significant reductions in WC, showing improvements after 12 weeks of ASE intervention [28,30]. Similarly, reported reductions in both the WC and BMI were reported following a 16-week ASE program [31].

These findings collectively establish ASE as a versatile and effective exercise intervention, offering significant benefits across multiple health domains. It plays a crucial role in reducing blood pressure, improving glucose metabolism, lowering inflammation, enhancing musculoskeletal function, and optimizing body composition. Given its accessibility and effectiveness, ASE presents a promising approach for individuals seeking to improve their overall health and prevent chronic diseases, as detailed in Table 1.

#### 3.3.1. Meta-Analysis

A meta-analysis was conducted of six distinct health outcomes including WC, HbA1c, FBG, HDL-C, SBP, and DBP.

#### 3.3.2. Effects of Arm Swing Exercise on WC

**Figure 2:**
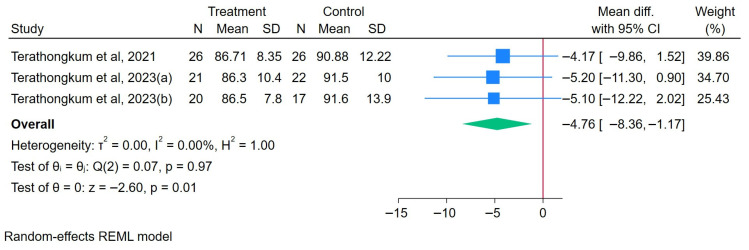
Forest plot of arm swing exercise on WC [28,30].

A total of 67 participants engaged in an ASE, while 65 were assigned to the control group. The results demonstrated that the ASE intervention significantly reduced the WC, with an overall MD of 4.76 (95% CI: −8.36 to −1.17), heterogeneity (I^2^) = 0.00%, and z = −2.60 (*p* = 0.01), as illustrated in Figure 2. A sensitivity analysis showed that the overall direction of effect remained stable after omitting each study, indicating that the pooled estimate was not overly influenced by any single study (Supplementary File S2A).

#### 3.3.3. Effects of Arm Swing Exercise on HbA1C

**Figure 3:**
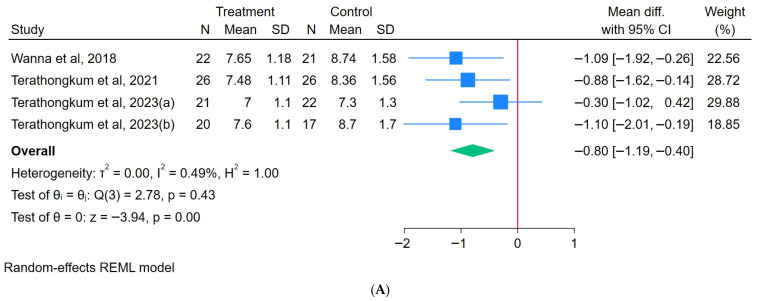

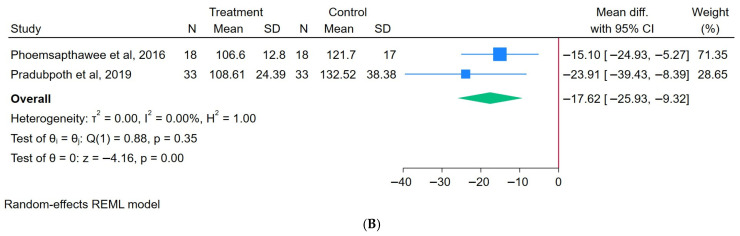
Forest plot of arm swing exercise on (**A**) HbA1c [27,28,30] and (**B**) FBG [23,25].

A total of 89 participants engaged in an ASE, while another 86 were assigned to the control group. The results showed that the ASE intervention significantly reduced the HbA1c, with an overall MD of −0.80 (95% CI: −1.19 to −0.40), heterogeneity (I^2^) = 0.00%, and z = −3.95 (*p* < 0.001), as illustrated in Figure 3A. A sensitivity analysis revealed consistent effect sizes and maintained statistical significance when either study was excluded, indicating that the findings were robust and not dependent on a single study (Supplementary File S2B).

#### 3.3.4. Effects of Arm Swing Exercise on FBG

A total of 51 participants engaged in an ASE, while another 51 were assigned to the control group. The results showed that the ASE intervention significantly reduced the FBG, with an overall MD of −17.62 (95% CI: −25.93 to −9.32), heterogeneity (I^2^) = 0.00%, and z = −4.16 (*p* < 0.001), as illustrated in Figure 3B. A sensitivity analysis demonstrated that the exclusion of either study did not affect the statistical significance or direction of the pooled effect size, confirming the robustness of the results (Supplementary File S2C).

#### 3.3.5. Effects of Arm Swing Exercise on HDL-C

**Figure 4:**
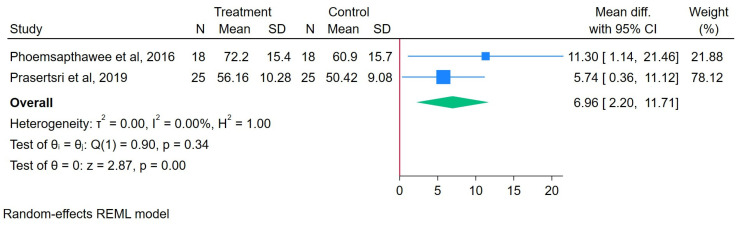
Forest plot of arm swing exercise on HDL-C [22,25].

A total of 43 participants engaged in an ASE, while another 43 were assigned to the control group. The results showed that the ASE intervention significantly increased the HDL-C, with an MD of 6.96 (95% CI: 2.20 to 11.71), heterogeneity (I^2^) = 0.00%, and z = 2.87 (*p* < 0.001), as illustrated in Figure 4. A sensitivity analysis showed that the direction and significance of the effect remained stable regardless of which study was removed, indicating the robustness of the pooled result (Supplementary File S2D).

#### 3.3.6. Effects of Arm Swing Exercise on SBP

**Figure 5:**
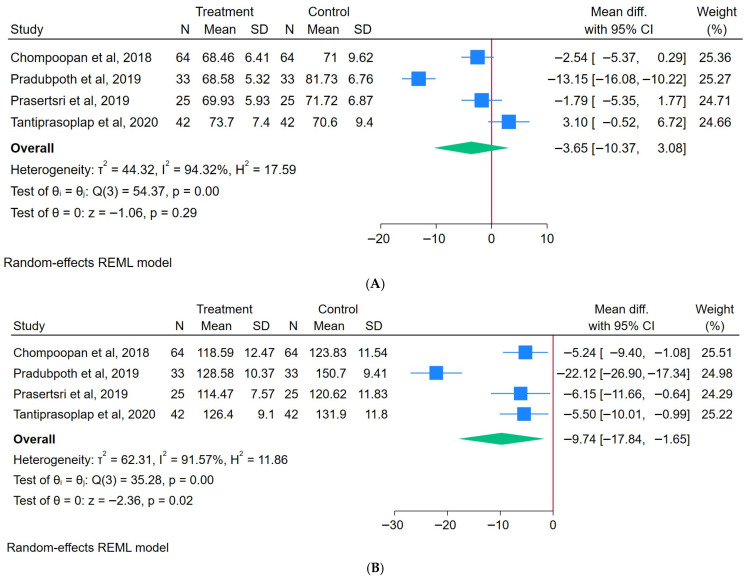
Forest plot of arm swing exercise on (**A**) SBP [13,22,23,24] and (**B**) DBP [13,22,23,24].

A total of 164 participants engaged in an ASE, while another 164 were assigned to the control group. The results showed that the ASE intervention produced a non-significant reduction in the SBP, with an overall MD of −3.65 (95% CI: −10.37 to 3.18), heterogeneity (I^2^) = 94.32%, and z = −1.05 (*p* = 0.29), as illustrated in Figure 5A. A sensitivity analysis showed that the exclusion of individual studies did not significantly change the effect size or its direction, indicating the findings were robust across the included studies (Supplementary File S2E).

#### 3.3.7. Effects of Arm Swing Exercise on DBP

A total of 97 participants engaged in an ASE, while another 97 were assigned to the control group. The results indicated that the addition of an ASE intervention did not result in a significantly reduced DBP, with an overall effect size (MD) of −9.74 (95% CI: −17.84 to −1.65), heterogeneity (I^2^) = 91.57%, and z = −2.36 (*p* = 0.02), as illustrated in Figure 5B. A sensitivity analysis showed consistent and statistically significant negative effect sizes across all leave-one-out scenarios, confirming the robustness of the findings (Supplementary File S2F).

## 4. Discussion

This systematic review and meta-analysis provided suggest that ASE is an effective intervention for enhancing multiple health domains, including cardiovascular health, metabolic control, inflammation reduction, musculoskeletal function, and body composition. ASE has been shown to be a simple yet impactful intervention, contributing to reductions in blood pressure, blood glucose levels, and inflammation markers, as well as improving in lipid profiles, cardiorespiratory fitness, and functional mobility. In addition, the meta-analysis demonstrates that ASE leads to significant reductions in the WC, DBP, HbA1c, and FBG, while also increasing HDL-C. Although the ASE showed a non-significant reduction in SBP, the overall trend was toward lower values. These outcomes suggest that ASE may be used as a viable exercise strategy for those at risk of metabolic syndrome, hypertension, and cardiovascular disease.

### 4.1. Effects on Waist Circumference

Our meta-analysis showed that the WC demonstrated one of the most pronounced improvements following an ASE, with a significant reduction of 4.76 cm. The studies included in this review demonstrated consistent improvements after 12 to 16 weeks of ASE training [28,30,31]. This finding highlights ASE’s potential in reducing abdominal fat, which is a critical marker of central obesity and an established risk factor for metabolic syndrome, T2DM, cardiovascular diseases, and mortality [32,33,34,35]. The substantial decrease in the WC observed aligns with existing evidence supporting the effectiveness of exercise in improving body composition and reducing visceral fat accumulation [36]. Given that a 5 cm reduction in the WC has been associated with a significantly lower risk of metabolic syndrome [37], these findings emphasize ASE’s effectiveness in targeting abdominal fat, reducing obesity-related health risks, and improving overall metabolic health.

### 4.2. Effects on Hemoglobin A1c and Fasting Blood Glucose

ASE demonstrated significant benefits in glycemic control. Glycemic control is a key clinical marker for the prevention and management of diabetes mellitus [38]. Our meta-analysis revealed a reduction in HbA1c levels by 0.80% and FBG levels by 17.62 mg/dL, particularly following 12-week ASE interventions [27,28]. These findings are in line with previous studies reporting significant improvements in HbA1c after structured exercise programs of a similar duration [39], reinforcing the role of ASE in promoting better glucose regulation. Moreover, evidence suggests that every 30 min per week of moderate-to-vigorous aerobic exercise can significantly improve HbA1c [40], highlighting the potential of even small increases in physical activity for meaningful glycemic improvements. Additionally, significant decreases in the FBG were observed following ASE interventions within as little as 5 to 12 weeks [23,25], suggesting that ASE may rapidly enhance glucose metabolism. Overall, these findings suggest that ASE may be effective in improving insulin sensitivity and glucose regulation. A 1% reduction in HbA1c levels has been associated with a 14% lower risk of myocardial infarction and more than a 20% reduction in overall mortality, as well as significant decreases in both microvascular and macrovascular complications [41,42,43]. Furthermore, they are consistent with prior research demonstrating that regular physical activity enhances glucose uptake, promotes insulin efficiency [44], and reduces the risk of diabetes-related complications [45]. Collectively, these findings underscore the potential of ASE as a practical and effective exercise strategy for enhancing glycemic control and reducing the risk of diabetes-related complications.

### 4.3. Effects on Cardiovascular Risk Factors

Our systematic review highlights the potential of ASE as a promising intervention for improving cardiovascular risk factors, particularly blood pressure, lipid profiles, and cardiorespiratory fitness. Notably, ASE interventions were associated with a non-significant mean reduction in SBP and a significant reduction in DBP, suggesting potential but not conclusive benefits for SBP and meaningful improvements in DBP in hypertension management. However, the observed effects on SBP and DBP exhibited high heterogeneity across studies, which may be attributed to variations in participant characteristics (e.g., age, baseline BP), intervention duration, and exercise intensity. This variability is consistent with the broader exercise literature, where meta-analyses have reported significant but heterogeneous reductions in SBP and DBP following aerobic and resistance training [8]. Despite this heterogeneity, the magnitude of DBP reduction remains clinically relevant. A reduction of just 5 mmHg in SBP is associated with a 10% lower risk of major cardiovascular events [46].

Our findings suggest that performing ASEs for 3 to 5 days per week over a period of 12 to 24 weeks leads to significant blood pressure reductions [13,17,22,23,24]. Recent studies have reinforced the role of exercise in reducing both systolic and diastolic blood pressure [47]. Additionally, a previous study highlighted that an 8–12-week aerobic exercise program can lead to significant reductions in 24 h ambulatory blood pressure among individuals with resistant hypertension. This suggests that even short-term exercise interventions can have meaningful impacts on blood pressure control [48]; the observed effects of ASE could have substantial clinical implications for preventing stroke, heart failure, and other cardiovascular complications, indicating lasting cardiovascular benefits.

Furthermore, ASE significantly increased HDL-C levels by 6.96 mg/dL, indicating potential benefits in lipid metabolism and cardiovascular protection. The increase in HDL-C suggests that rhythmic upper-body movement and moderate aerobic activity can enhance cholesterol transport and fat oxidation, reducing the risk of atherosclerosis and coronary artery disease [17,22]. These findings align with existing research indicating that moderate-intensity aerobic exercise can reduce systemic inflammation and promote anti-inflammatory responses [49,50,51].

In addition, our synthesis found that ASE significantly improves cardiorespiratory fitness, as evidenced by increases in the VO_2_ peak and exercise capacity [25,26], which are well-established indicators of cardiovascular endurance. These findings align with previous studies demonstrating that exercise enhances vascular function, oxygen utilization, and overall heart health [52,53], suggesting enhanced aerobic fitness and cardiovascular efficiency. Given that a higher VO_2_ peak is associated with reduced all-cause mortality, these findings emphasize the importance of ASE in improving long-term physical resilience and mobility

ASE significantly enhances the musculoskeletal function, particularly in muscle strength, endurance, balance, and flexibility. Studies have demonstrated that ASE improves neuromuscular coordination, postural stability, and movement efficiency, which are crucial for reducing fall risks and improving overall mobility, especially in older adults. Our systematic review reported significant gains in muscle strength, flexibility, balance, gait, and cognitive function, supporting ASE’s role in functional movement enhancement [8,29]. These findings suggest that ASEs may be particularly beneficial for elderly populations, individuals recovering from musculoskeletal impairments, and those at risk of age-related mobility decline.

Also, the increase in high-frequency power and decrease in low-frequency power suggest that ASE positively influences autonomic nervous system regulation [13], contributing to reduced stress levels and improved relaxation responses. This aligns with previous studies indicating that resistance exercise is the most effective method for enhancing muscle strength in older adults, particularly those experiencing sarcopenia and physical frailty [54,55].

In summary, while heterogeneity in blood pressure outcomes warrants cautious interpretation, the overall evidence supports ASE as a multifaceted intervention with significant implications for cardiovascular health, especially in aging and sedentary populations.

### 4.4. Comparative Effectiveness of ASE Across Health Outcomes in Meta-Analysis

The effect size (ES) is an important measure in meta-analysis, allowing us to compare the magnitude of ASE’s impact across different health outcomes. By analyzing the effect sizes of ASE on WC, HbA1c, SBP, FBG, HDL, and DBP, we can determine which outcome the ASE influences the most and rank its effectiveness accordingly.

By comparing effect sizes, it is evident that ASE has the greatest impact on the WC (ES = 1.20) and HbA1c (ES = 1.02), highlighting its role in obesity reduction and long-term glucose control. The effects on SBP (ES = 0.87) and FBG (ES = 0.64) suggest moderate improvements in cardiovascular and metabolic function, while its influence on HDL (ES = 0.56) and DBP (ES = 0.45) is smaller. These findings suggest that ASE is most effective in reducing abdominal fat and improving long-term glucose regulation, making it a valuable exercise for managing metabolic syndrome, obesity, and diabetes. While its effects on blood pressure and lipid metabolism are beneficial, they are more moderate and may require additional lifestyle modifications to achieve greater clinical significance.

Although a comprehensive search strategy without country restrictions was employed, all studies included in this review were conducted in Thailand following the health promotion policy, which may limit the generalizability of the findings to broader populations and diverse settings. At the same time, this geographic concentration underscores the novelty of ASE as a culturally specific intervention that has received limited investigation outside Thailand. In addition, the relatively small number of high-quality randomized controlled trials and final review articles currently available represents a key limitation, as it restricts the strength of the evidence base and the ability to perform more detailed subgroup or sensitivity analyses. In contrast, in many Western countries, a greater emphasis is placed on lifestyle-integrated physical activities such as walking, stair climbing, or aerobic exercise, which are commonly recommended by healthcare providers for chronic disease prevention and healthy aging [56,57]. Given the widespread burden of physical inactivity and the increasing prevalence of non-communicable diseases worldwide, particularly in low- and middle-income countries, interventions such as ASE offer practical benefits by overcoming barriers related to cost, equipment, and accessibility. Moreover, considerable variability in ASE protocols across studies, including differences in session duration, intensity, and execution, should be considered, as it may limit the development of standardized recommendations and highlights the need for future stratified analyses to identify optimal practices. Another limitation is that the underlying mechanisms of ASE remain insufficiently explained, and unmeasured confounding factors such as concurrent lifestyle changes or behavioral influences may have contributed to the observed outcomes. In addition, potential publication and language biases should be acknowledged, as unpublished studies and non-English or Thai literature were not included, which may have led to an overestimation of the effects. Furthermore, direct comparisons between ASE and other forms of exercise such as walking, aerobic exercise, and resistance training are scarce, limiting conclusions on its relative effectiveness and adherence.

Future research should aim to address these limitations by conducting larger-scale, multicenter trials across diverse populations and cultural contexts, as well as by performing rigorous meta-analyses once sufficient evidence accumulates. Comparative effectiveness studies that directly evaluate ASE against other widely practiced exercises could clarify its relative value in terms of health outcomes, adherence, and cost-effectiveness. Furthermore, mechanistic investigations are warranted to elucidate the physiological pathways underlying ASE’s benefits, thereby strengthening the scientific rationale for its application. Finally, building international collaborations and incorporating ASE into global health promotion strategies would help determine its scalability and sustainability as a low-cost, accessible intervention.

The integration of ASE into public health initiatives may provide an innovative and scalable solution to enhance physical activity levels globally. Given the global diversity in physical activity patterns, future research should address these gaps by comparing ASE with other commonly practiced forms of exercise in diverse sociocultural contexts. In addition, these findings underline the importance of incorporating ASE into professional training programs across medicine, nursing, physical therapy, and physical education. Equipping health professionals with the knowledge and skills to apply ASE in prevention, health promotion, and rehabilitation contexts could facilitate its broader implementation and maximize the public health impact.

## 5. Conclusions

This meta-analysis provides compelling evidence that ASE is an effective intervention for improving cardiovascular function, glycemic control, body composition, and physical performance. The observed reductions in DBP, HbA1c, FBG, and WC, along with increases in HDL-C and aerobic fitness, suggest that ASE can serve as a practical and sustainable strategy for promoting long-term health and preventing chronic diseases. Although the ASE demonstrated a non-significant reduction in SBP, the overall trend supports its potential benefit for systolic blood pressure. However, the high heterogeneity observed in blood pressure outcomes highlights the need for more standardized protocols and consistent reporting in future studies. To enhance the reliability and generalizability of findings, future research should incorporate larger sample sizes, longer follow-up periods, and stratified analyses based on participant characteristics and intervention parameters. Addressing these methodological variations will be crucial for optimizing ASE recommendations and integrating them effectively into public health interventions.

## Figures and Tables

**Table 1:** Characteristics of included studies.

Study	Country	Design	Participants		Intervention	Duration (WK)	Outcome
			Age (Year)	Sample (Intervention/Control)			
1. Saelao et al., 2012 [29]	Thailand	Quasi-experimental	60–69 yrs	63 (15/17/15)	ASE 3 days per week and 50 min a day	8	Muscular strength and endurance ↑ Flexibility of the body ↑ Balance ↑ BMI Fat mass VO_2_ max %Fat Lean mass
2. Phoemsapthawee et al., 2016 [25]	Thailand	RCT	65–87 yrs	36 (18/18)	ASE 5 days per week; 20 min/day during weeks 1–3, 25 min/day in week 4, and 30 min/day from week 5 onward, with warm-up/cool-down and progression in swing speed	12	FBG ↓ HDL↑ VO_2_ peak ↑ TG TC LDL-C
3. Prasertsri et al., 2017 [26]	Thailand	RCT	21.22 ± 2.84 yrs	60(20/20/20)	ASE 3 days per week and 30 min a day	8	Exercise Capacity↑ VO_2_ peak↑ Resting HR
4. Wanna et al., 2018 [27]	Thailand	RCT	20–59 yrs	43(22/21)	ASE 5 days per week and 30 min a day, with diabetes education and follow-up support	12	HbA1c ↓ WC BMI
5. Prasertsri et al., 2018 [17]	Thailand	RCT	71.80 ± 7.16 yrs	51 (25/26)	ASE 3 days per week and 30 min a day	12	SBP ↓ PP ↓ W/H ↓ hsCRP ↓ LF power ↓ HDL ↑ HF power ↑ K^+^ Mg^2+^
6. Chompoopan et al., 2018 [13]	Thailand	RCT	>60 yrs	128 (64/64)	ASE 5 days per week and 30 min a day, with 5 min warm-up and 5 min cool-down	24	SBP ↓ DBP ↓ HR ↓ Body balance ↑
7. Prasertsri et al., 2019 [22]	Thailand	RCT	60–68 yrs	50 (25/25)	ASE 3 days per week and 30 min a day; 1-month follow-up assessed for carry-over effects	12	SBP ↓ hsCRP ↓ HDL ↑ K^+^ ↑ Mg^2+^ ↑ LF power HF power
8. Pradubpoth et al., 2019 [23]	Quasi-experimental research design.	Quasi-experimental	>60 yrs	66 (33/33)	ASE 5 days per week and 15 min a day	12	SBP ↓ DBP ↓ FBG ↓
9. Tantiprasoplap et al., 2020 [24]	Thailand	RCT	>60 yrs	84 (42/42)	ASE 3 days per week and 30–40 min a day, combined with low sodium intake education	12	SBP ↓ DBP HR VO_2max_
10. Terathongkum et al., 2021 [28]	Thailand	Quasi-experimental	>20 yrs	76 (26/24/26)	ASE 5 days per week and 30 min a day	12	HbA1c ↓ WC↓ BMI WC Visceral fat Skeletal muscle
11. Terathongkum et al., 2023 [30]	Thailand	Quasi-experimental	>18 yrs	80(41/39)	ASE 5 days per week and 30 min a day	12	WC↓ HbA1c BMI VF Visceral fat Skeletal muscle
12. Kiewdee et al., 2023 [16]	Thailand	Quasi-experimental	18–60 yrs	52 (26/26)	ASE 130 min a day	16	WC↓ BMI ↓
13. Xiao et al., 2023 [8]	Thailand	RCT	68.3 + 5.6 yrs	56 (28/28)	ASE 5 days per week and 40 min a day	8	Posture ↑ Flexibility ↑ Gait ↑ Cognition ↑

ASE: arm swing exercise, FBG: fasting blood glucose, HDL: high-density lipoprotein cholesterol, VO_2_ peak: peak oxygen consumption, TG: Triglyceride, TC: Total Cholesterol, HbA1c: glycated hemoglobin, SBP: systolic blood pressure, PP: pulse pressure, W/H: waist-to-hip ratio, hsCRP: High-Sensitivity C-Reactive Protein, LF Power: Low-Frequency Power, HF Power: High-Frequency Power, HR: Heart Rate, K^+^: potassium, Mg^2+^: magnesium, DBP: diastolic blood pressure, WC: waist circumference, BMI: body mass index, and VO_2max_: cardiorespiratory fitness, ↑: increase; ↓: decrease.

## Supplementary Materials

The following supporting information can be downloaded at: https://www.mdpi.com/article/10.3390/healthcare13182357/s1, Supplementary File S1. Quality appraisal; Supplementary File S2A: Sensitivity analyses for the effect size of WC; Supplementary File S2B: Sensitivity analyses for the effect size of HBA1C; Supplementary File S2C: Sensitivity analyses for the effect size of FBG; Supplementary File S2D: Sensitivity analyses for the effect size of HDL-C; Supplementary File S2E: Sensitivity analyses for the effect size of SBP; Supplementary File S2F: Sensitivity analyses for the effect size of DBP.
