# Supplementary material for: Effectiveness of Arm Swing Exercise on Comprehensive Health Outcomes: A Systematic Review and Meta-Analysis
Source: Healthcare (Basel). 2025 Sep 19;13(18):2357. doi: 10.3390/healthcare13182357 (PMC12469779; doi:10.3390/healthcare13182357)
Supplement: Supplementary file 1 [file healthcare-13-02357-s001.zip › healthcare-3810893-supplementary.pdf]

Supplementary file

1. **Supplementary Table 1.** Quality appraisal

Table 1. Quality appraisal

JBI Critical Appraisal of Eligible Quasi-Experimental Study

Studies	*Q1*	*Q2*	*Q3*	*Q4*	*Q5*	*Q6*	*Q7*	*Q8*	*Q9*
Saelao et al 2012	*Y*	*U*	*U*	*Y*	*Y*	*Y*	*Y*	*Y*	*Y*
Pradubpoth et al, 2019	*Y*	*Y*	*Y*	*Y*	*Y*	*Y*	*Y*	*Y*	*Y*
Terathongkum et al, 2021	*Y*	*Y*	*Y*	*Y*	*Y*	*Y*	*Y*	*Y*	*Y*
Terathongkum et al, 2023	*Y*	*Y*	*Y*	*Y*	*Y*	*Y*	*Y*	*Y*	*Y*
Kiewdee et al, 2023	*Y*	*Y*	*Y*	*Y*	*Y*	*Y*	*Y*	*Y*	*Y*

JBI Critical Appraisal of Eligible Randomized Controlled Trial

Studies	*Q1*	*Q2*	*Q3*	*Q4*	*Q5*	*Q6*	*Q7*	*Q8*	*Q9*	*Q10*	*Q11*	*Q12*	*Q13*
Phoemsapthawee et al, 2016	*Y*	*U*	*Y*	*N*	*N*	*U*	*Y*	*Y*	*Y*	*Y*	*Y*	*Y*	*Y*
Prasertsri et al, 2017	*Y*	*U*	*Y*	*N*	*N*	*U*	*Y*	*U*	*Y*	*Y*	*Y*	*Y*	*Y*
Wanna et al, 2018	*Y*	*U*	*Y*	*N*	*N*	*U*	*Y*	*Y*	*Y*	*Y*	*Y*	*Y*	*Y*
Prasertsri et al, 2018	*Y*	*U*	*Y*	*N*	*N*	*U*	*Y*	*Y*	*Y*	*Y*	*Y*	*Y*	*Y*
Chompoopan et al, 2018	*Y*	*U*	*Y*	*N*	*N*	*U*	*Y*	*Y*	*Y*	*Y*	*Y*	*Y*	*Y*
Prasertsri et al, 2019	*Y*	*U*	*Y*	*N*	*N*	*U*	*Y*	*Y*	*Y*	*Y*	*Y*	*Y*	*Y*
Tantiprasoplap et al, 2020	*Y*	*Y*	*Y*	*N*	*N*	*Y*	*Y*	*Y*	*Y*	*Y*	*Y*	*Y*	*Y*
Xiao et al, 2023	*Y*	*Y*	*Y*	*N*	*N*	*U*	*Y*	*Y*	*Y*	*Y*	*Y*	*Y*	*Y*

Y: Yes; N: No; U: Unclear; NA: Not applicable

JBI Critical Appraisal of Eligible Quasi-Experimental Study

1. Is it clear in the study what is the 'cause' and what is the 'effect' (i.e. there is no confusion about which variable comes first)?
2. Were the participants included in any comparisons similar?
3. Were the participants included in any comparisons receiving similar treatment/care, other than the exposure or intervention of interest?
4. Was there a control group?
5. Were there multiple measurements of the outcome both pre and post the intervention/exposure?
6. Was follow up complete and if not, were differences between groups in terms of their follow up adequately described and analyzed?
7. Were the outcomes of participants included in any comparisons measured in the same way?
8. Were outcomes measured in a reliable way?
9. Was appropriate statistical analysis used?

JBI Critical Appraisal of Eligible Randomized Controlled Trial

1. Was true randomization used for assignment of participants to treatment groups?
2. Was allocation to treatment groups concealed?
3. Were treatment groups similar at the baseline?
4. Were participants blind to treatment assignment?
5. Were those delivering treatment blind to treatment assignment?
6. Were outcomes assessors blind to treatment assignment?
7. Were treatment groups treated identically other than the intervention of interest?
8. Was follow-up complete and if not, were differences between groups in terms of their follow up adequately described and analyzed?
9. Were participants analyzed in the groups to which they were randomized?
10. Were outcomes measured in the same way for treatment groups?
11. Were outcomes measured in a reliable way?
12. Was appropriate statistical analysis used?
13. Was the trial design appropriate, and any deviations from the standard RCT design (individual randomization, parallel groups) accounted for in the conduct and analysis of the trial?

Supplementally file 2

Sensitivity analyses for the Arm Swing Exercise on WC

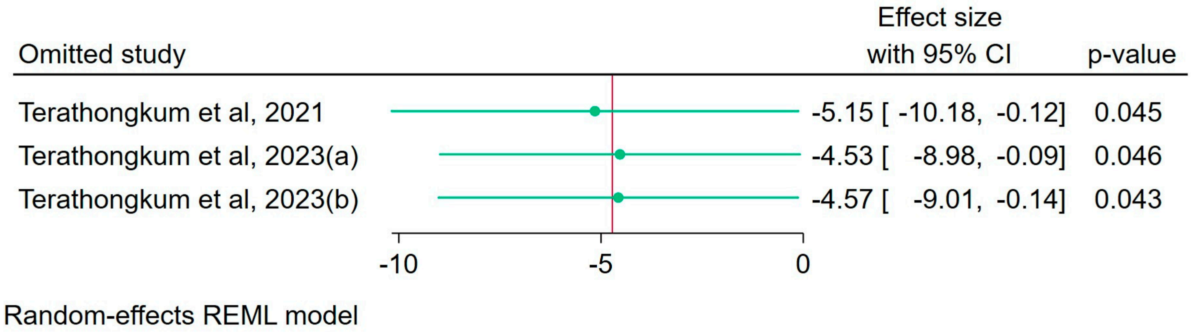

Supplemental 2A: Sensitivity analyses for the effect size of WC

Sensitivity analyses for the Arm Swing Exercise on HBA1C

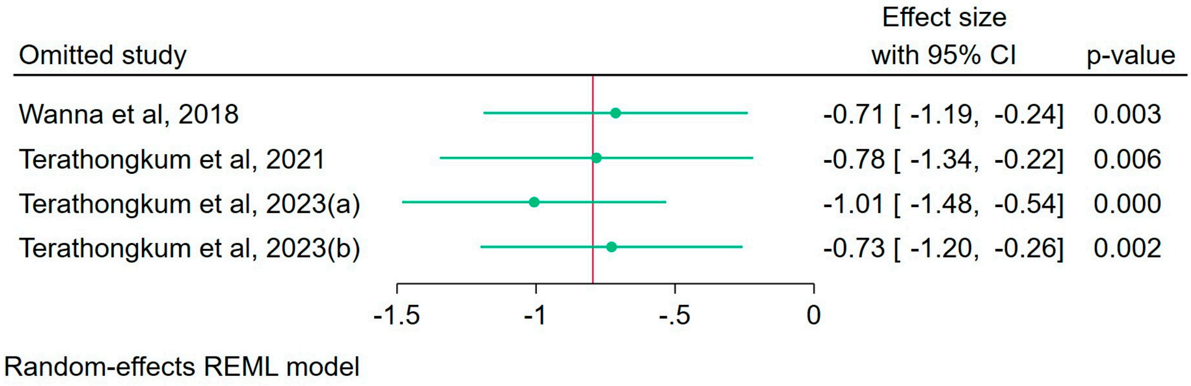

Supplemental 2B: Sensitivity analyses for the effect size of HBA1C

Sensitivity analyses for the Arm Swing Exercise on FBG

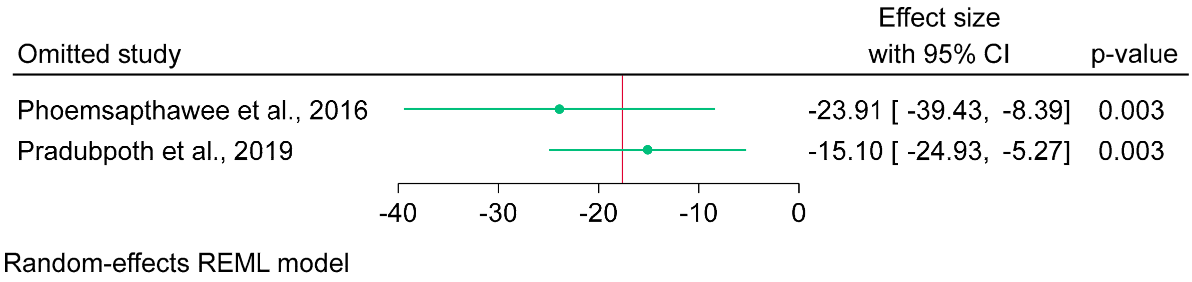

Supplemental 2C: Sensitivity analyses for the effect size of FBG

Sensitivity analyses for the Arm swing exercise on HDL-C

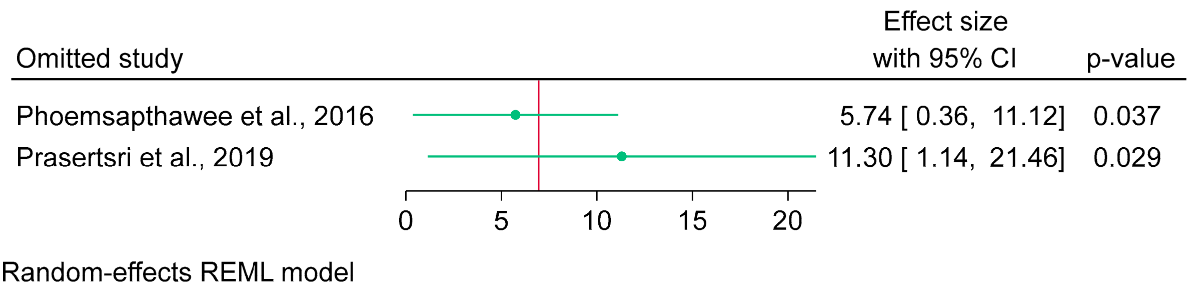

Supplemental 2D: Sensitivity analyses for the effect size of HDL-C

Sensitivity analyses for the Arm Swing Exercise on SBP

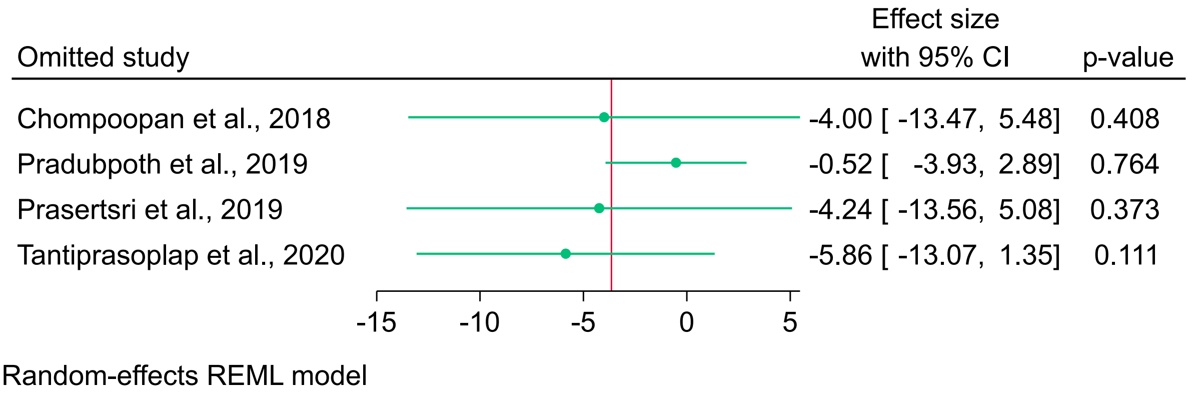

Supplemental 2E: Sensitivity analyses for the effect size of SBP

Sensitivity analyses for the Arm Swing Exercise on DBP

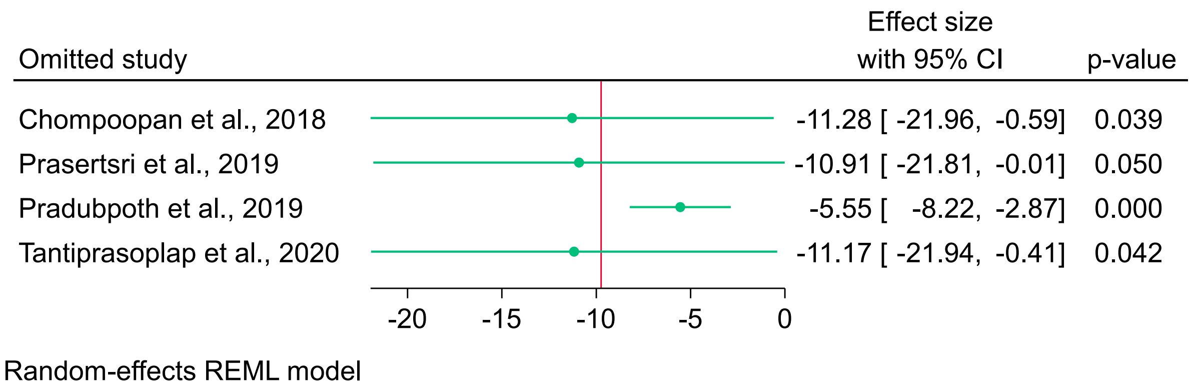

Supplemental 2F: Sensitivity analyses for the effect size of DBP
